# Evaluation of the Impacts of Marine Salts and Asian Dust on the Forested Yakushima Island Ecosystem, a World Natural Heritage Site in Japan

**DOI:** 10.1007/s11270-012-1297-z

**Published:** 2012-09-12

**Authors:** Takanori Nakano, Yoriko Yokoo, Masao Okumura, Seo-Ryong Jean, Kenichi Satake

**Affiliations:** 1 Research Institute for Humanity and Nature, 457-4 Kamigamo Motoyama, Kita-ku, Kyoto, 603-8047 Japan; 2 Department of Environmental Systems Science, Doshisha University, 1-3 Tataratuya, Kyotanabe, Kyoto 610-0331 Japan; 3 Japan Oil, Gas and Metals National Corporation, 1310 Omiya, Saiwai-ku, Kawasaki, 212-8554 Japan; 4 Department of Geoscience, Chonbuk National University, 664-1, 567-756 Jeonju, Jeollabukdo South Korea; 5 Faculty of Geo-environmental Science, Rissho University, 1700 echi, Kumagaya, Saitama 360-0194 Japan

**Keywords:** Yakushima, Asian dust, Stream water, Chemical weathering, Sr isotope, Nd isotope, Ca depletion

## Abstract

To elucidate the influence of airborne materials on the ecosystem of Japan’s Yakushima Island, we determined the elemental compositions and Sr and Nd isotope ratios in streamwater, soils, vegetation, and rocks. Streamwater had high Na and Cl contents, low Ca and HCO_3_ contents, and Na/Cl and Mg/Cl ratios close to those of seawater, but it had low pH (5.4 to 7.1), a higher Ca/Cl ratio than seawater, and distinct ^87^Sr/^86^Sr ratios that depended on the bedrock type. The proportions of rain-derived cations in streamwater, estimated by assuming that Cl was derived from sea salt aerosols, averaged 81 % for Na, 83 % for Mg, 36 % for K, 32 % for Ca, and 33 % for Sr. The Sr value was comparable to the 28 % estimated by comparing Sr isotope ratios between rain and granite bedrock. The soils are depleted in Ca, Na, P, and Sr compared with the parent materials. At Yotsuse in the northwestern side, plants and the soil pool have ^87^Sr/^86^Sr ratios similar to that of rainwater with a high sea salt component. In contrast, the Sr and Nd isotope ratios of soil minerals in the A and B horizons approach those of silicate minerals in northern China’s loess soils. The soil Ca and P depletion results largely from chemical weathering of plagioclase and of small amounts of apatite and calcite in granitic rocks. This suggests that Yakushima’s ecosystem is affected by large amounts of acidic precipitation with a high sea salt component, which leaches Ca and its proxy (Sr) from bedrock into streams, and by Asian dust-derived apatite, which is an important source of P in base cation-depleted soils.

## Introduction

The atmosphere of the Japanese archipelago is rich in marine aerosols from the surrounding ocean and has been adversely affected by acidic pollutants and dust minerals transported from the Asian continent (Hatakeyama et al. 2004; Shimizu et al. 2004; Inoue et al. 2005; Nakano et al. 2006; Seto et al. 2007; Hartmann et al. 2008). Monitoring studies over more than 10 years have shown the acid rain impact on soil and aquatic ecosystems in the mountainous area of Japan (e.g., Kurita and Ueda 2006; Nakahara et al. 2010). However, few researchers have evaluated the impacts of atmospheric deposition of continental-derived materials on Japan’s terrestrial and aquatic ecosystems. The effects of rain and aerosols on biogeochemical cycles are so complex that an integrated approach that considers entire ecological systems as single interacting units is required to understand these effects. Nutrients and other ions in the soil–vegetation system and in terrestrial water are ultimately derived not only from the atmosphere but also from weathering of the soil and the underlying bedrock. Accordingly, identification and quantification of atmosphere- or bedrock-derived materials in plants, soils, and streamwater are important for assessing the biogeochemical cycles in terrestrial ecosystems.

Rainwater in Japan has ^87^Sr/^86^Sr ratios that clearly differ from those of the substrate rocks at depositional sites, and it contains high quantities of Sr and Ca that are derived from acid-soluble minerals (mainly calcium carbonate) that originated in the desert and loess areas of northern China (Nakano and Tanaka 1997; Nakano et al. 2006). Sr is a good proxy for Ca (Miller et al. 1993; Ǻberg 1995; Clow et al. 1997), which is essential for plant growth (as are K, P, and Si), and the ^87^Sr/^86^Sr ratios of water and vegetation are affected by the ratios present in a basin’s bedrock (Graustein 1988; Faure and Mensing 2005). The ^87^Sr/^86^Sr ratio and concentrations of dissolved ions in rainwater show temporal variation (Nakano and Tanaka 1997; Nakano et al. 2006), whereas those of a stream’s base flow are temporally invariant and can therefore be considered to represent year-round water characteristics (Rose and Fullagar 2005). Accordingly, Sr isotopes have been utilized as powerful tracers for determining the sources and flows of Ca within soil–vegetation systems (e.g., Miller et al. 1993; Ǻberg 1995; Blum et al. 2002) and aquatic systems (e.g., Clow et al. 1997; Shand et al. 2009). Nd isotopes also have considerable potential as atmospheric and environmental tracers, since the soils in northern China are reported to have ^143^Nd/^144^Nd ratios (*ε*_Nd_ values) that are distinct from those of many rocks in Japan (Bory et al. 2003; Nakano et al. 2004). Several Sr and Nd isotope studies have shown that Asian dust minerals are deposited in the soils of southwestern Japan (Mizota et al. 1992) and Hawaii (Chadwick et al. 1999; Kurtz et al. 2001), but few studies have used both isotopes as biogeochemical tracers in terrestrial systems (Pett-Ridge et al. 2009).

**Fig. 1:**
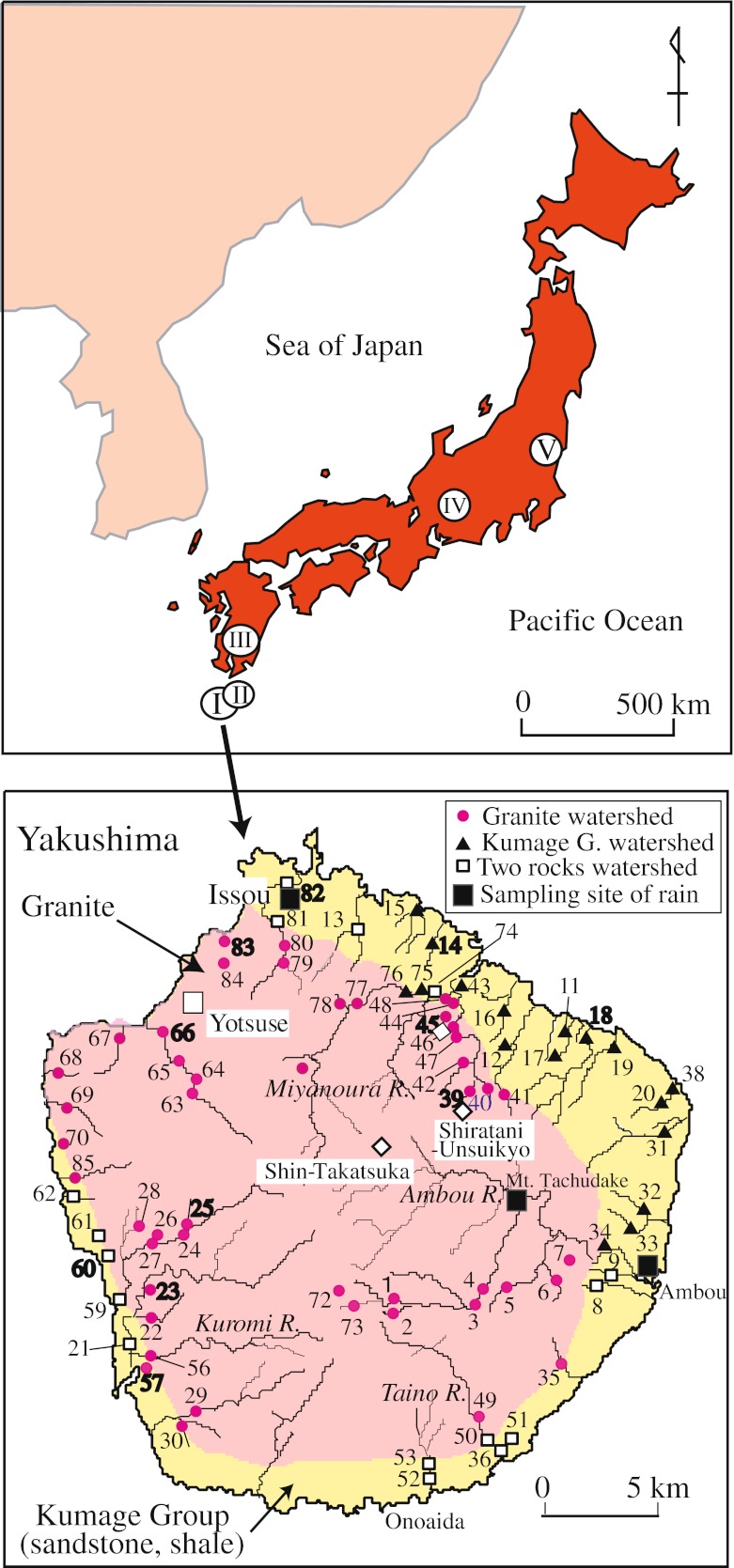
*Upper* *map* location of Yakushima Island and the study sites: *I*, Yakushima Island; *II*, Tanegashima; *III*, northern Kagoshima; *IV*, Naegi; and *V*, Tsukuba. *Bottom map* sampling sites locations and geological background of Yakushima. *Red circles*, *black triangles*, and *empty squares* represent streamwater sampling points in areas with bedrock dominated by granite, bedrock dominated by sedimentary rocks of the Kumage group, and mixtures of the two types of rocks, respectively. The *large empty square* indicates the Yotsuse sample site discussed in the text. Three *large filled squares* indicate the locations of the rainwater monitoring and sampling by Tamaki et al. (1991), Satake et al. (1998), and Nakano et al. (2000). Sampling locations in areas with granitic bedrock are 23, 39, 45, 57, 66, and 83, and those in areas with Kumage sedimentary rocks are 14, 60, and 82. The sites where both streamwater and bedrock were collected are shown in *boldface*. *Diamonds* indicate the sampling sites of soil in Nakano et al. (2001c)

Yakushima Island, in southwestern Japan (Fig. 1), became a world natural heritage site in 1993 in recognition of its unique and irreplaceable forested ecosystem. This island faces the Asian continent across the East China Sea, and rainfall and some tree (*Pinus amamiana*) on the island are intensely affected by aerosols from the surrounding sea and by acidic materials, including gases (SOx and NOx) and aerosols, transported from China (Satake et al. 1998; Nakano et al. 2000; Nagafuchi et al. 2001; Kume et al. 2010). The annual average pH of rain on Yakushima is 4.7, a value equivalent to that on the main islands of Japan (Tamaki et al. 1991; Japan Environmental Sanitation Center 2002). However, the mean annual precipitation on Yakushima ranges from 2,500 to 4,700 mm at lower altitudes along the coast, and it exceeds 8,600 mm in mountainous areas (Eguchi 1984). These amounts are three to five times the precipitation on the main Japanese islands, indicating that Yakushima is receiving proportionally higher total inputs of acidic materials in precipitation. Further, the geology of Yakushima is widely composed of granite, which is known to have small acid neutralization capacity. Nevertheless, the impact of rain and dust minerals from the Asian continent on the island’s plants, soils, and streamwater is unclear. This study was undertaken to elucidate the geochemical and Sr and Nd isotopic characteristics of Yakushima’s aquatic, soil, and vegetation systems and their responses to these atmospheric inputs.

## Study Site and Methodology

### Geography and Geology

Yakushima Island is located 70 km south of Kyushu (30° N, 130° E), Japan’s third largest island. This small island, 132 km in circumference and 503 km^2^ in area, consists of steep mountains covered by dense natural forests with many cliffs and with many waterfalls owing to the large amounts of precipitation. Mt. Miyanoura, the highest point on the island, at 1,935 m above sea level (a.s.l.), is also the highest peak in the Kyushu region. The annual mean temperature is around 20 °C at the coast; this corresponds to the margin between the subtropical and warm temperate zones (Tagawa 1994); however, the temperature decreases with increasing elevation, and areas above 1000 m a.s.l. receive snow in winter. Accordingly, there are distinct altitudinal zones of vegetation. About 14,000 residents live in small areas of Yakushima, mostly along the coast at elevations less than 100 m a.s.l.

The island is composed mainly of Miocene granites of the ilmenite series that contain orthoclase megacrysts with maximum lengths of 14 cm, as well as plagioclase, quartz, and biotite, with small amounts of chlorite, apatite, zircon, tourmaline, muscovite, and ilmenite (Sato and Nagashima 1979). Anma et al. (1998) classified Yakushima’s granite into four types on the basis of its occurrence, texture, and petrochemistry: the Yakushima main granite, the core granodiorite, the core cordierite granodiorite, and the core cordierite granite. The Yakushima main granite occupies 90 % of the total area of the Yakushima pluton, whereas the other granites are locally distributed. The Yakushima granite body is an intrusion within the Kumage Group, which originated in the Paleogene age and is composed mainly of sandstone and shale distributed around the periphery of the island. These sedimentary materials are sometimes overlain unconformably by terrace deposits, talus deposits, and Quaternary alluvium, mainly along the eastern and southern coasts. A pyroclastic flow deposit called Akahoya covers these rocks in some areas.

### Samples

There are three sites (Issou, Tachudake, and Ambou in Fig. 1) for monitoring the precipitation chemistry in Yakushima. Detailed compositional data are available for the Issou site, where rainwater was collected with a bulk sampler at intervals of 1 or 2 weeks from 1994 to 1996. This site is about 250 m above sea level and 5 m from the ground, on top of a building, and trees, as viewed from the sampler, cover less than 30° of the sky (i.e., there is little or no interference from trees).

From 1996 to 1997 we sampled streamwater at 79 locations chosen on the basis of their basin geology during the baseflow period from summer to autumn (Fig. 1). These samples were divided into three groups: those in granite-dominated watersheds, those in watersheds dominated by the Kumage sedimentary rock, and those in watersheds that include both types of rock. For comparison of streamwater quality in relation with the watershed geology, we sampled streamwater from several areas with a range of geological conditions and with negligible upstream human activity on Tanegashima Island, which is close to Yakushima and composed primarily of sedimentary rock; in the northern part of Kagoshima Island, which is composed primarily of granitic rock, sedimentary rock, and volcanic rock (mostly andesitic); in the Naegi area of Chubu district, which is composed mostly of granite; and in the Tsukuba area of Ibaraki prefecture, which is composed mostly of granite and gabbro (Fig. 1). At each site, the water samples were filtered through disposable cellulose acetate filters with a pore size of 0.2 μm; pH and alkalinity were measured immediately after sampling.

We also collected eight granite samples at six locations and four samples of the Kumage sedimentary rocks at four locations (Fig. 1). Soil is well developed on the hills and gentle slopes. At the Yotsuse site in the northwestern part of Yakushima, facing the Asian continent (Fig. 1), we collected samples of three plant species and soil samples at seven depths. This site is located at the top of a small hill (200 m a.s.l.), where the granitic bedrock is deeply weathered to produce horizons in the soil profile; the thicknesses of the A horizon and the B horizon were 30 and 170 cm, respectively, whereas the C horizon reached a depth of more than 500 cm.

### Analysis

We dried about 40 g of soil from each horizon overnight at 105 °C in an oven. The dried samples were then reacted with 10 % *v*/*v* hydrogen peroxide (H_2_O_2_) solution in a tall beaker at 70 °C to separate the organic fraction. The solution was then centrifuged (Kokusan Enshinki, H-103N Series) at 2,400 rpm for 30 min. The supernatant was used for the Sr isotope analysis. The residual fraction was washed with ultrapure water; after centrifugation for 30 min, this supernatant was then discarded. We collected residual soils after repeating this rinse procedure three times. Three soil fractions (<2, 2 to 20, and >20 μm) were separated from about 10 g of the residual soil by means of Stokes’ law gravity sedimentation in deionized water. They were then concentrated by centrifugation. Bulk soils and these fractions were digested with a solution of HF, HClO_4_, and HNO_3_. We also extracted soil samples of about 0.5 g with 1 N acetic acid (HOAc) solution to remove the exchangeable fraction. The remaining solution was used for the Sr isotope analysis. Rock samples were pulverized in a tungsten carbide vessel with a HERZOG HSM-F36 disk mill (HERZOG Automation Corp., Osnabrück, Germany) to obtain powdered samples for chemical and Sr isotope analysis. All reagents used in this leaching and dissolution procedure were of analytical grade or better.

Chemical analyses were performed at the Chemical Analysis Center and the Institute of Geoscience, University of Tsukuba. The concentrations of cations and anions in streamwater were determined by means of inductively coupled optical emission spectrometry (Jarrell Ash ICAP-757V, Kyoto, Japan) and a Yokokawa Analytical Systems (Yokogawa, Japan) IC7000 ion chromatograph, respectively. The chemical compositions of the rocks and soils were determined by means of X-ray fluorescence with a Phillips PW1404 analyzer. We determined Sr and Nd isotope ratios by using a Finnigan MAT 262RPQ mass spectrometer at the University of Tsukuba and a Thermo Fisher TRITON mass spectrometer at the Research Institute for Humanity and Nature. The mean ^87^Sr/^86^Sr ratio of nine standard NBS987 samples during this study was 0.710246 (2*σ*_mean_, ±0.000022; *n* = 9) using the MAT262 RPQ and 0.710278 (2*σ*_mean_, ±0.000012; *n* = 5) using the TRITON, and all measurements were normalized with respect to the recommended ^87^Sr/^86^Sr ratio of 0.710250. The ^143^Nd/^144^Nd ratio of the La Jolla standard was 0.511846 ± 0.000011 (2*σ*_mean_, *n* = 12).

## Results and Discussion

### Streamwater System

#### Geochemical Characteristics of Yakushima Streamwater

**Fig. 2:**
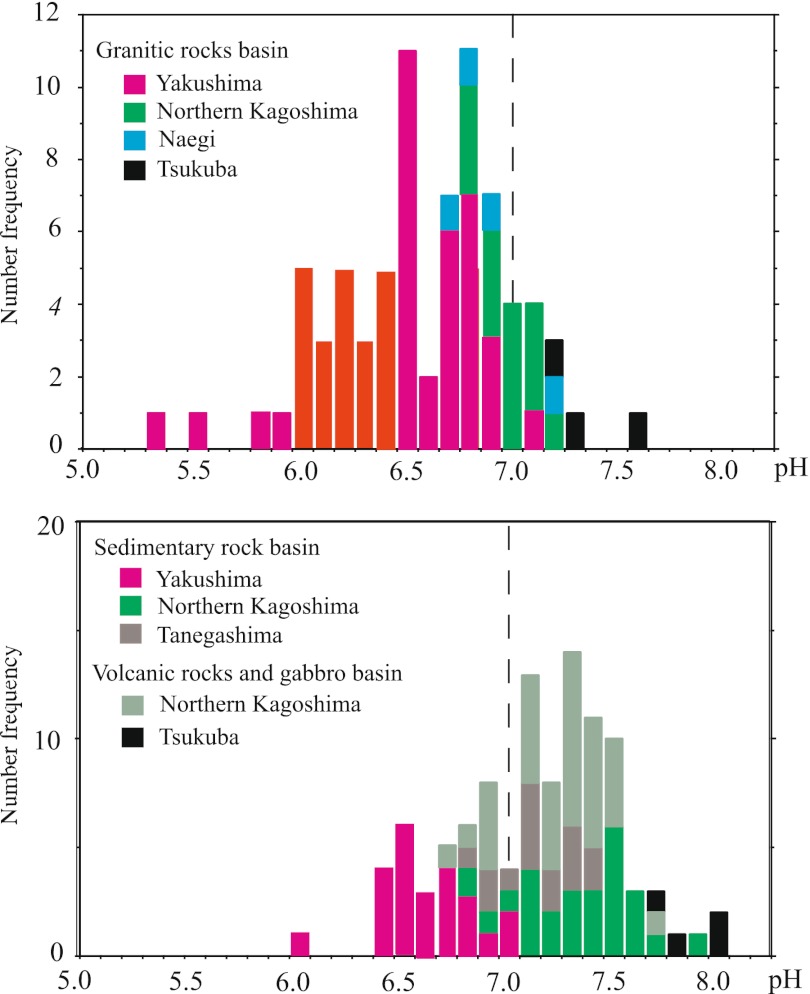
Frequency distribution of streamwater pH in (*upper graph*) watersheds with granitic bedrock and (*lower graph*) watersheds with bedrock from the Kumage series of clastic sedimentary rocks, with volcanic rocks (mostly andesitic), and with gabbro. *Vertical dashed lines* represent neutral pH (7.0)

Streamwater was classified into three types based on the geology of the upstream watershed of the sampling point. The chemical compositions of dissolved ions in the streamwater of Yakushima (Table 1) showed a large geographical variation, but did not differ significantly between the samples from watersheds with granitic bedrock and those with Kumage Group bedrock. The mean water quality values for streamwater in Yakushima for the two type’s watershed geology and those from the other study areas are summarized in Table 2. Streamwater from all areas except Yakushima was neutral to slightly alkaline, but there was a tendency for the streamwater in granitic watersheds to be slightly more acidic than those in watersheds with sedimentary or volcanic rock (Fig. 2); the average pH (±*σ*_mean_) values for streamwater in the granitic watershed (III, IV, and V in Table 2) and in watersheds with sedimentary or volcanic rock (II, III, and V in Table 2) were 6.87 ± 0.28 and 7.26 ± 0.27, respectively.

**Table 1:** Chemical composition and Sr isotopic ratios of water from individual streams in accordance with the watershed geology in Yakushima Island

No.	Elevation (m)	EC (μS/cm)	Temp (°C)	pH	^87^Sr/^86^Sr	F^−^	Cl^−^	NO_3_^−^	SO_4_^2−^	HCO_3_^-^	Na^+^	K^+^	Ca^2+^	Mg^2+^	Si	Sr^2+^	Ba^2+^
						μeqmol L^−1^										neqmol L^−1^	
Granite watersheds																	
1^a^	1190	16.4	9.0	6.00	0.70840	4	141	1	25	26	132	21	37	35	75	201	82
2^a^	1220	18.5	9.2	6.58	0.70832	3	139	0	21	68	123	9	56	38	93	247	73
3^a^	1100	17.4	9.7	5.86	0.70863	4	161	1	28	29	120	9	36	44	52	187	114
4^a^	950	20.0	11.6	6.55	0.70831	3	156	1	26	44	151	9	41	36	121	219	98
5^a^	840	16.6	11.8	6.19	0.70852	3	131	5	27	28	120	10	28	32	72	158	102
6^a^	665	16.8	12.0	5.50	0.70874	4	114	2	45	12	119	6	26	32	24	151	70
7^a^	555	19.6	12.4	6.19	0.70861	4	139	3	41	32	131	9	40	37	47	208	76
22^a^	190	51.6	13.6	6.82	0.70830	3	369	3	62	92	347	23	89	81	181	342	73
23^a^	270	57.6	13.1	6.37	0.70833	3	453	25	72	40	389	22	103	103	158	388	90
24	515	42.9	13.9	6.82	0.70818	2	281	3	53	116	325	20	85	64	233	336	67
25	510	28.9	10.9	6.50	0.70849	3	207	6	45	44	186	11	52	48	102	205	55
26	540	36.6	11.5	6.65	0.70843	3	262	10	60	60	250	15	69	54	146	263	63
27	540	36.5	13.2	6.41	–	2	309	16	61	35	289	12	67	66	147	263	64
28	640	38.1	11.7	6.51	0.70827	3	252	6	63	76	275	16	79	54	192	285	63
29^a^	120	42.1	14.0	6.75	0.70846	3	306	4	59	80	295	17	84	64	155	295	57
30^a^	120	68.0	13.7	6.92	0.70854	3	517	6	74	116	499	28	134	108	245	491	70
35^a^	220	45.4	16.3	7.13	0.70847	2	271	39	49	64	256	19	84	76	140	283	80
39^a^	625	30.6	13.1	6.70	0.70848	3	214	3	39	68	225	18	55	47	177	251	111
40^a^	670	27.1	11.5	6.49	0.70853	3	225	2	37	104	214	14	40	50	133	226	102
41^a^	680	26.7	11.1	6.53	0.70864	2	209	6	41	48	215	14	47	46	154	233	80
42^a^	580	25.5	11.0	6.25	0.70858	3	211	5	46	40	195	8	43	48	115	210	76
44	205	–	–	6.89	–	3	253		80	124	308	17	104	59	262	418	42
45	185	–	–	6.87	–	3	273	4	78	104	324	19	85	64	235	356	58
46	200	–	–	6.41	–	3	266		60	36	257	11	58	58	129	247	64
47	215	–	–	6.83	0.70828	2	258		59	88	283	19	70	56	212	304	71
48	40	–	–	6.23	–	3	236	5	51	44	221	11	56	53	117	240	73
49^a^	255	34.8	14.6	6.06	0.70862	3	286	7	49	32	267	17	45	61	122	221	83
56^a^	30	30.9	15.3	6.61	0.70843	3	216	5	46	20	213	13	60	50	144	249	66
57^a^	70	54.2	14.4	6.95	0.70846	2	421	10	69	140	410	23	111	93	236	461	84
63	95	33.4	15.4	6.53	0.70842	3	226	5	58	44	234	16	71	51	148	276	64
64	95	33.5	14.5	6.70	0.70840	3	227	5	58	66	232	16	71	51	147	279	68
65	85	42.6	14.3	6.70	0.70822	3	289	15	72	84	310	21	105	69	216	393	74
66	10	32.1	15.1	6.54	0.70842	4	216	6	58	60	225	14	71	51	137	272	64
67	10	42.8	15.6	6.45	0.70843	3	287	8	72	68	303	19	97	66	189	354	70
68	145	74.3	14.8	6.37	0.70842	4	576	31	115	44	534	35	141	124	209	555	112
69	130	56.5	14.1	6.01	0.70856	4	478	21	107	20	440	31	114	108	144	461	134
70	140	83.0	14.5	6.18	0.70850	4	726	21	142	44	649	46	172	161	169	683	124
72^a^	1630	16.2	7.8	5.36	0.70844	3	146	1	26	8	104	9	22	39	39	189	50
73^a^	1385	15.9	8.6	6.50	0.70847	3	135		21	28	98	10	37	38	66	235	50
77	150	33.8	13.6	6.48	0.70835	4	241	6	62	47	224	14	61	56	137	308	35
78	205	33.4	14.5	6.30	0.70830	3	235	11	55	40	216	15	57	55	139	304	45
79	190	40.3	13.4	6.72	0.70829	3	273	6	67	40	270	17	87	63	183	379	34
80	130	37.1	13.2	6.70	0.70836	3	255	2	60	100	255	17	72	59	163	349	44
83	70	50.1	14.1	6.80	0.70876	3	339		72	108	357	21	95	74	244	479	45
84	135	44.6	13.1	6.87	0.70871	3	327		67	108	334	19	81	70	228	434	45
85	175	56.9	12.9	6.07	0.70875	5	475	17	99	8	388	18	109	104	123	475	87
Average		37.3	12.9	6.47	0.70846	3	277	8	58	59	268	17	73	63	150	312	73
Kumage group watersheds (sedimentary rock)																	
11	65	45.5	15.8	6.68	0.71204	4	298	9	73	84	284	17	91	87	135	372	86
12	245	33.9	15.6	6.64	0.71236	3	218	13	52	48	220	14	51	64	138	306	63
14	50	52.7	15.6	6.83	0.71345	3	357	11	98	96	385	37	123	87	161	495	71
15	40	54.3	15.0	6.51	0.71231	4	332	12	109	80	347	23	107	84	142	477	73
16	105	39.3	15.6	6.52	0.71023	3	272	13	58	40	262	21	50	74	133	313	73
17	130	38.0	14.4	6.43	0.71218	3	260	10	60	40	227	17	65	69	100	292	61
18	15	37.8	15.6	6.58	0.71202	3	258	12	59	44	241	22	64	68	115	290	52
19	65	44.0	15.5	6.62	0.71288	3	325	5	57	68	292	30	67	82	137	352	70
20	140	46.3	16.3	6.72	0.71234	3	336	3	53	84	306	24	69	89	144	393	93
31	110	45.3	16.3	6.58	0.71269	2	263	14	53	68	265	23	66	78	135	370	82
32	120	41.1	16.7	6.75	0.71246	2	249	5	48	68	245	18	61	72	146	320	61
33	115	44.7	16.6	6.78	0.71337	2	260	10	51	92	260	23	78	76	165	370	52
34	160	38.8	17.3	6.72	0.71179	2	242	2	39	48	217	16	47	65	124	244	54
38	35	80.4	15.3	6.51	0.71041	5	727	7	107	52	654	29	87	169	126	450	101
43	40	47.6	15.9	6.87	0.71254	3	312	7	46	116	319	28	110	93	194	452	80
75	75	39.4	14.1	6.40	0.71296	3	274	5	76	52	251	20	80	75	143	406	51
76	100	35.6	14.3	6.44	0.71070	3	242	6	69	48	229	13	55	64	136	363	51
Average		45.0	15.6	6.75	0.71216	3	307	9	65	66	294	22	75	82	140	369	69
Mixing watersheds with granite and Kumage group sedimentary rock																	
8	270	23.2	14.8	6.05	0.70932	4	163	1	53	12	149	14	39	42	23	178	77
9	180	37.6	17.1	6.84	0.71108	3	229	7	58	116	228	17	109	73	102	370	86
13	85	43.8	14.9	6.53	0.70851	4	327	11	113	60	354	26	88	82	166	505	66
21	20	25.7	12.2	6.53	–	4	183	2	41	32	163	10	44	44	75	183	50
36	65	21.5	16.0	6.40	0.70871	3	119	5	32	16	121	10	32	32	76	128	57
50	140	20.9	14.0	5.97	0.70853	3	142	4	32	24	138	11	36	35	93	167	63
51	65	21.9	14.3	6.07	0.70880	3	147	5	34	32	144	10	38	37	95	174	61
52	70	28.0	15.0	6.22	0.70869	3	195	5	57	32	204	21	47	44	86	217	84
53	190	26.0	14.2	6.25	0.70866	4	183	5	60	2	179	10	44	44	81	187	133
59	30	33.8	15.6	6.82	0.70846	3	242	5	50	64	209	13	59	52	142	247	60
60	50	112.7	17.1	7.05	0.70882	6	678	14	146	304	855	48	191	162	400	728	60
61	60	34.4	17.3	6.28	0.70842	3	233	8	50	36	219	16	52	53	139	242	102
62	290	97.2	15.4	6.50	0.70933	4	959	11	137	48	819	42	188	182	196	715	102
74	50	29.5	13.3	6.04	0.70871	3	211	5	50	40	197	14	56	52	120	295	51
81	50	38.7	13.7	6.90	0.70834	4	270	4	62	68	255	16	75	61	166	361	44
82	10	49.3	14.2	6.54	0.70836	3	337	1	72	92	334	21	89	81	185	418	50
Average		41.7	14.8	6.53	0.70864	4	302	6	67	61	299	19	74	68	144	326	70
Overall average		39.7	14.0	6.54	0.70940	3	286	8	61	61	277	18	73	68	145	326	72

Sample numbers refer to the locations shown in Fig. 1

*EC* conductivity

^a^The site of granite watershed in northwestern side

**Table 2:** Average of electric conductivity (EC), pH, and concentrations of dissolved ions in stream waters from drainage basins with different bedrock types and those of rain on Yakushima and seawater

Bedrock in the basin (area)		Sample number	EC (μS cm^−1^)	pH	Si	Cl^−^	SO_4_^2−^	NO_3_^−^	HCO_3_^−^	Ca^2+^	Mg^2+^	Na^+^	K^+^	Sr^2+^	Na/Cl	Mg/Cl	Ca/Cl	K/Cl	Sr/Cl ×10^3^	Ca/Na	K/Na	Mg/Na	Ca/K
					μmol L^−1^																		
I	Granite (Yakushima)	55	37	6.45	150.3	277.0	29.1	8.4	58.0	36.4	31.3	267.9	16.9	0.16	0.97	0.11	0.13	0.06	0.56	0.14	0.06	0.12	2.16
	Sedimentary rock (Yakushima)	24	45	6.62	139.9	307.8	32.6	8.5	66.4	37.2	41.2	294.5	22.0	0.18	0.96	0.13	0.12	0.07	0.60	0.13	0.07	0.14	1.69
I^a^	Granite BDC									24.7	5.4	52.2	10.8							0.47	0.21	0.10	2.29
	Kumage BDC									24.1	12.8	54.7	15.2							0.44	0.28	0.23	1.59
II	Sedimentary rock (Tanegashima)	15	111	7.14	232.9	680.7	107.1	48.1	433.0	178.7	158.0	726.8	52.1	0.62	1.07	0.23	0.26	0.08	0.91	0.25	0.07	0.22	3.43
III	Granite (N. Kagoshima)	14	50	7.04	309.8	167.6	52.5	11.6	233.3	126.8	46.1	224.9	26.6	0.45	1.34	0.28	0.76	0.16	2.66	0.56	0.12	0.20	4.77
	Sedimentary rock (N. Kagoshima)	25	92	7.38	380.6	189.3	145.3	48.4	554.6	267.7	130.9	368.0	78.0	0.99	1.94	0.69	1.41	0.41	5.25	0.73	0.21	0.36	3.43
	Volcanic rock (N. Kagoshima)	35	93	7.25	628.8	223.7	99.5	42.9	485.7	223.3	96.4	370.8	78.9	0.58	1.66	0.43	1.00	0.35	2.60	0.60	0.21	0.26	2.83
IV	Granite (Naegi)	4	26	6.88	199.0	41.5	22.2	8.1	130.0	45.2	9.1	129.6	22.8	0.15	3.13	0.22	1.09	0.55	3.58	0.35	0.18	0.07	1.98
V	Granite (Tsukuba)	3	76	7.39	459.3	215.5	38.6	46.6	383.6	130.7	43.6	428.9	31.5	0.52	1.99	0.20	0.61	0.15	2.44	0.30	0.07	0.10	4.16
	Gabbro (Tsukuba)	4	150	7.91	478.9	215.0	46.9	58.5	1109.7	398.2	170.0	417.6	34.3	0.88	1.94	0.79	1.85	0.16	4.09	0.95	0.08	0.41	11.62
Average of rain at Yakushima^b^		–	37	4.70	–	157.2	27.7	16.3	–	6.7	14.7	122.5	3.45	–	0.78	0.09	0.04	0.02	–	0.05	0.03	0.12	1.94
Seawater^c^		–	–	–	–	545,952	28,250	–	1,967	10,280	53,086	468,465	10,205	90.16	0.86	0.10	0.02	0.02	0.17	0.02	0.02	0.11	1.01

Numbers I to V represent the locations shown in Fig. 1

^a^Average concentration of cations in Yakushima streamwater contributed by the bedrock, granite and Kumage group

^b^Rain water data are after Nakano et al. (2000)

^c^Seawater values are those presented by Berner and Berner (1987)

This difference is consistent with the composition of granite, which is composed mainly of minerals that are resistant to chemical weathering (i.e., quartz and potassium feldspar) and that thus have a lower capacity to buffer acids in the rain. One remarkable feature is that the Yakushima streamwater was more acidic than that in the other basins, with pH ranging from 5.4 to 7.1 (an average of 6.5) versus a range of 6.7 to 8.0 at the other sites. Furthermore, the streamwater at the other sites was generally a CaHCO_3_ or NaHCO_3_ type, whereas the Yakushima streamwater was generally a NaCl type. The average Na and Cl concentrations in the Yakushima streamwater were about 7.6 and 4.7 times the average Ca and HCO_3_ concentrations, respectively.

Monthly analysis of the rainwater composition at the Issou site (Satake et al. 1998; Nakano et al. 2000) revealed that the concentrations of the major dissolved ions were high in winter and low in summer, but that the proportions of Na, Mg, and Cl (Fig. 2 in Nakano et al. 2000) were roughly constant throughout the year and were almost identical to those in seawater, indicating that these three ions are largely of sea salt origin. The non-sea salt (NSS) Ca and K fractions in the Yakushima rainwater were 0.6 ± 0.2 and 0.3 ± 0.2, respectively (Fig. 4 in Nakano et al. 2000). Nakano et al. (2000) suggested from their Sr isotope study that the NSS Ca is derived mainly from plant cover on Yakushima that dominantly contains Sr with a marine isotopic signature. Table 2 provides the mean pH, electrical conductivity, and concentrations of the main ions in precipitation.

**Fig. 3:**
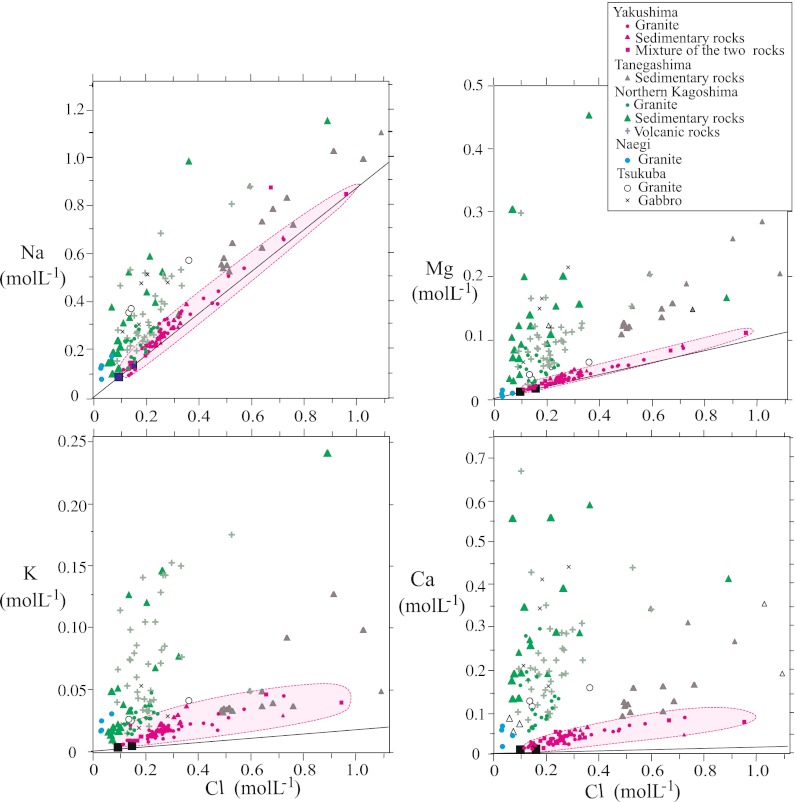
Concentrations of the four major cations (Na, Mg, K, Ca) as functions of the Cl concentration in streamwater on Yakushima and in other areas with different bedrock geologies. *Solid lines* indicate values based on the ratios in seawater (Berner and Berner 1987). The *two filled squares* represent the mean compositions in rainwater at two sites [elevations of 475 m (high Cl) and 40 m (low Cl)] on Yakushima in a study by Tamaki et al. (1991)

Chloride is assumed to be a conservative tracer for the input of sea salt aerosols (Berner and Berner 1987), and the ratio of a given cation to the Cl concentration in streamwater therefore increases as a result of addition of the cation to soil water through chemical weathering. The concentrations of the major cations (Na, K, Ca, and Mg) in Yakushima streamwater were positively correlated with the Cl concentration (Fig. 3). In addition, the Na/Cl and Mg/Cl ratios of the Yakushima streamwater were close to those of seawater (Table 2). Although the Ca/Cl and K/Cl ratios of the Yakushima streamwater were considerably higher than those of seawater (Table 2), the values were still closer to the seawater ratios than to those of streamwater from other areas of Japan. These results strongly suggest that the acidic precipitation on Yakushima contains a substantial sea salt component, which in turn controls the chemical composition of dissolved elements in the Yakushima streamwater.

**Fig. 4:**
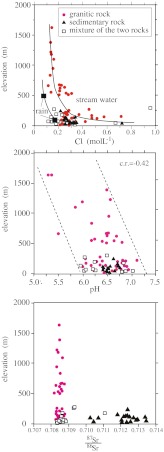
Concentration of Cl (*top*), pH (*middle*), and ^87^Sr/^86^Sr ratios (*bottom*) in Yakushima streamwater as a function of elevation. Most streamwater samples from granite watersheds are from small drainage areas, and this can minimize the effects of the elevation at which the rainwater falls. The concentrations of Cl in rainwater at two sites on Yakushima (*large filled squares*) were taken from Tamaki et al. (1991)

The sea salt component of the rain generally decreases with increasing distance from the coast (Berner and Berner 1987). Tamaki et al. (1991) reported the average elemental composition of wet precipitation over 2 years at two Yakushima sites with different altitudes, Ambou at 40 m a.s.l. and Tachudake at 475 m a.s.l. (Fig. 1). They found that rainfall on Yakushima had a lower annual average Cl concentration at 475 m than at 40 m, but had the same annual average pH value (4.7). The concentration of Cl in the streamwater of Yakushima, where the watershed is small, tended to decrease with elevation (Fig. 4). At several low-elevation sites, the Cl content of the streamwater was very high (>0.5 mol L^−1^). Because of the high humidity that results from the heavy rainfall in the study area, this high Cl content in streamwater cannot be explained only by the concentration process that results from the evaporation of rainwater; instead, it suggests the dry deposition of sea spray in areas near the shore.

The positive correlations of cations in Yakushima streamwater with the Cl concentration indicate that the cation concentrations tend to decrease with elevation. The altitudinal decreases of Cl and cation concentrations in streamwater are likely to be caused by the increased contribution of rainfall at higher elevations, which increases the amount of water relative to the amount of sea salt. The correlation coefficient between pH and elevation for the Yakushima streamwater is −0.42 (*P* < 0.05), suggesting that, although the correlation is weak, streamwater at high elevations tends to be more acidic (Fig. 4). A similar pattern of decreasing streamwater pH with elevation has been observed in base-poor watersheds at the Hubbard Brook Experimental Forest (New Hampshire, USA) that have been affected by inputs of acidic deposition (Palmer et al. 2005). Accordingly, the altitudinal decrease of Yakushima streamwater pH value may be partly ascribed to the effects of the larger amount of acidic rain because the mountainous area receives a high input of H^+^ ions from the atmosphere, and these cannot be fully neutralized by weathering of the granitic bedrock.

**Fig. 5:**
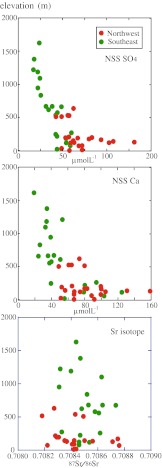
Altitudinal change of concentration of NSS SO_4_ (*top*), NSS Ca (middle), and ^87^Sr/^86^Sr ratios (*bottom*) of streamwater in the granite watershed between the northwestern and southeastern sides

NSS sulfate (NSS SO_4_) is an important component responsible for the formation of acid rain. Nakano et al. (2001a) and Ebise and Nagafuchi (2002) reported that streamwater in the northwestern side of Yakushima island contained higher concentration of NSS SO_4_ than that in the southeastern side (Fig. 5), and attributed this areal variation of streamwater to acidic deposition transported from the northwestern Asian continent. However, the concentration of non-sea salt cations such as NSS Ca in streamwaters of the granite watershed did not show a meaningful difference between the northwestern and southeastern sides compared to their altitudinal decreases. This result suggests that the chemical weathering of granite is affected by the amount of precipitation and other acids such as carbonic and/or organic acids generating in soil–vegetation system rather than the atmosphere-derived NSS SO_4_.

#### Rainwater Contribution to Cations in Streamwater

When there is no direct contribution from human activity, the ions dissolved in the streamwater should ultimately originate from the atmosphere and from the watershed’s bedrock. In other words, dissolved ions in streamwater as well as in the soil water can be separated into an atmosphere-derived component and a bedrock-derived component (BDC), which can be subclassified into granite BDC for the BDC from the granites and Kumage BDC for the BDC from the Kumage sedimentary rocks. It is generally assumed that Cl in streamwater is derived mainly from precipitation when there is no volcanic gas or evaporites (both of which are enriched in Cl) in the watershed (Berner and Berner 1987; Nakano et al. 2001b; Négrel et al. 2005). On the other hand, bedrock is generally considered to be the major source of cations in streamwater through chemical weathering. The concentrations of dissolved ions in water increase as a result of evaporation, but their ratios are generally assumed not to change substantially. Accordingly, the relatively constant ratios of dissolved cations to Cl in Yakushima streamwater indicate that the concentrations of the individual cations derived from bedrock through chemical weathering increase at a relatively constant rate with decreases in the sea salt component derived through the atmosphere.

If all Cl in the Yakushima streamwater is derived from a sea salt component dissolved in rainwater and/or sea spray, and if the uptake of elements by vegetation is negligible, then the proportion of these elements in the streamwater that is derived from rainwater (*f*_rain_) can be calculated for a dissolved cation (*X*, representing Na, K, Ca, and Mg) by using the following equation:

1 $$ {f_{\text{rain}}} = {{{{{\left( {{{X} \left/ {\text{Cl}} \right.}} \right)}_{\text{rainwater}}}}} \left/ {{{{\left( {{{X} \left/ {\text{Cl}} \right.}} \right)}_{\text{streamwater}}}}} \right.} $$

Using the annual average of the compositions of dissolved ions in Yakushima rainwater (Table 2), we calculated that 32 % of the Ca and 36 % of the K in the streamwater of watersheds with granitic bedrock were derived from precipitation, whereas the rainwater contributed 81 % of the Na and 83 % of the Mg. In other words, two thirds of the Ca and K in the streamwater originated from weathering of the granites and Kumage sedimentary rock. The average order of dominance of the cations in streamwater contributed by the granite BDC was Na (52.2 μmol L^−1^) > Ca (24.7 μmol L^−1^) > K (10.8 μmol L^−1^) > Mg (5.4 μmol L^−1^). The order for the Kumage BDC was Na (54.7 μmol L^−1^) > Ca (24.1 μmol L^−1^) > K (15.2 μmol L^−1^) > Mg (12.8 μmol L^−1^; Table 2).

#### Effects of Chemical Weathering of Granite on the Yakushima Streamwater

As mentioned above, the chemical composition of the streamwater did not differ significantly between watersheds with granite bedrock and Kumage sedimentary bedrock (Table 2). This result can be ascribed to the similarity of the dissolved ions in the BDC for both bedrocks. For example, the average molar ratios of Ca, K, and Mg to Na in the streamwater for the granite BDC (0.47, 0.21, and 0.10) were generally similar to those in the streamwater for the Kumage BDC (0.44, 0.28, and 0.23). On the other hand, Table 3 shows corresponding ratios of 0.32 for Ca/Na, 0.91 for K/Na, and 0.19 for Mg/Na from the granites, versus 0.32, 0.83, and 0.62, respectively, for the Kumage sedimentary rock, showing that both rocks have similar Ca/Na and K/Na ratios but very different Mg/Na ratios.

**Table 3:** Elemental compositions, Sr isotopic ratios, and *ε*_Nd_ values of granite and Kumage sedimentary rock in Yakushima and loess in China

Sampling site number	Si	Ti	Al	M	Mn	Mg	Ca	Na	K	P	Rb	Sr	Zr	Nd	Ca/Na	K/Na	Mg/Na	Ca/K	^87^Sr/^86^Sr	^87^Sr/^86^Sr*	*ε*_Nd_
	wt%									ppm					Molar ratio						
Granite																					
Granite (avg.)	33.15	0.33	8.08	2.35	0.05	0.52	1.55	2.39	3.20	603	183	154	165	26	0.373	0.788	0.204	0.474	0.70826	0.70758	-4.12
23	29.24	0.28	8.21	2.04	0.04	0.44	0.99	2.22	6.17	598	246	200	103	–	0.255	1.635	0.188	0.156	0.70810	0.70763	
39	31.05	0.31	7.49	2.22	0.05	0.51	1.72	2.68	3.20	546	173	180	131	–	0.368	0.701	0.180	0.525	0.70818	0.70762	
45	30.75	0.32	7.76	2.25	0.04	0.52	1.47	2.53	3.74	506	169	147	115	–	0.333	0.868	0.193	0.383	0.70827	0.70761	
57	30.98	0.24	7.94	1.70	0.04	0.38	1.68	2.71	3.80	515	161	196	127	–	0.356	0.824	0.134	0.431	0.70810	0.70740	
66	31.78	0.25	7.83	1.82	0.05	0.41	1.55	2.71	3.60	476	207	154	129	–	0.328	0.779	0.141	0.421	0.70817	0.70774	
66	30.36	0.31	7.84	2.20	0.05	0.51	1.23	2.64	4.86	580	216	204	113	–	0.267	1.083	0.181	0.247	0.70814	0.70760	
66	30.96	0.31	7.77	2.61	0.06	0.68	1.06	2.26	3.11	576	157	180	89	–	0.270	0.810	0.287	0.334	0.70821	0.70755	
83	31.98	0.31	7.18	2.16	0.05	0.51	1.58	2.59	2.98	519	161	143	125	–	0.350	0.675	0.184	0.518	0.70818	0.70767	
Average	31.14	0.29	7.79	2.15	0.05	0.50	1.43	2.53	3.85	547	186	173	122	26	0.322	0.907	0.188	0.388	0.70818	0.70760	
Kumage sedimentary rock																					
14	38.73	0.22	5.00	2.11	0.02	0.77	0.84	1.61	0.74	305	42	118	162	–	0.300	0.270	0.453	1.113	0.71504		−7.52
60	29.44	0.34	8.76	3.02	0.04	1.14	0.96	1.34	3.81	393	160	198	170	–	0.412	1.669	0.803	0.247	0.71606		
82	33.20	0.25	6.63	2.92	0.05	1.34	0.95	2.19	2.01	349	85	253	121	–	0.249	0.540	0.579	0.461	0.71548		
Average	33.79	0.27	6.79	2.68	0.04	1.08	0.92	1.71	2.19	349	96	190	151	–	0.320	0.826	0.612	0.607	0.71553		
Chinese loess																					
Bulk loess		0.32	5.75	2.75	0.06	1.25	4.81	1.27	1.72	818	87	231	86	28	2.180	0.799	0.936	2.729	0.71572		−10.31
Loess-1		0.39	6.47	3.07	0.04	1.09	0.66	1.40	1.83	591	133	136	117	27	0.269	0.768	0.739	0.351	0.71927		−10.79
Loess-2		0.39	5.70	1.02	0.02	0.49	0.62	1.52	1.70	108	109	143	133	19	0.233	0.657	0.302	0.354	0.71979		−11.19

The “Granite (avg.)” values in the first line represents the average values for 14 major Yakushima granites (Anma et al. 1998). Chinese loess values represent the average of six loess samples from northen China from Yokoo et al. (2004). ^87^Sr/^86^Sr* represents the estimated initial ^87^Sr/^86^Sr values at an age of 14 Ma. Sampling site numbers corresponded to the sites listed in Table 1 and Fig. 1. Loess-1 and loess-2 are the minerals after 5 % HOAc leaching and 20 % HCl leaching of loess, respectively

This difference can be ascribed to the chemical weathering process because the cation compositions differed remarkably between the BDC in streamwater and in the bedrock. One notable feature is that the molar Ca/K ratios of the granite BDC (2.28) and of the Kumage BDC (1.58) were much higher than those of the corresponding rocks (0.39 and 0.61, respectively). This contrast is attributable to the higher susceptibility of Ca minerals than K minerals to chemical weathering; thus, the bedrock would become progressively depleted of Ca at a faster rate than K.

The mineralogical composition of the granite is more distinct than that of the sandstone and shale in the Kumage sedimentary rock. The major cations in the streamwater of the granite BDC can be largely ascribed to the dissolution of potassium feldspar for K and Na, of plagioclase for Ca and Na, and of biotite for K and Mg. Potassium is the dominant cation in the Yakushima granite but is present at a lower level in the granite BDC. As the modal composition of K feldspar and plagioclase in the granite is around 30 % respectively, whereas that of biotite is about 5 % (Anma et al. 1998), this result shows that the potassium feldspar does not supply enough K into water.

In addition to plagioclase, the Ca in granite is substituted as accessory minerals such as apatite and carbonates. The average Ca content of apatite in the Yakushima granite is estimated to be 1,180 ppm on the basis of the P content (an average of 547 ppm; Table 3). Although previous studies have not described the presence of carbonates in the Yakushima granite, White et al. (2005) showed that most granite in the world contains at least some carbonates, with an average modal ratio of 0.25 %. If the Yakushima granite contains 0.25 % carbonates, as estimated by White et al. (2005), the Ca concentration in the carbonates would be 0.1 wt%. On the other hand, the mean Ca content of the Yakushima granite was 1.43 wt% (Table 3). Accordingly, the total amount of Ca derived from apatite and carbonates in the Yakushima granite would be less than 15 %. According to Nakano et al. (2001c), the soils on Yakushima are depleted in Ca compared with the original granite parent material, with the depletion reaching more than 90 % in the C horizon, which is composed primarily of weathered granite. This result indicates that the Ca in the granite BDC can be attributed mainly to weathering of the plagioclase.

Although plagioclase and potassium feldspar are two mineral sources of Na in the streamwater, the average molar ratio of K to Ca in the granite BDC of streamwater (0.44) is very low compared with that in the granite (2.97), indicating that the major source of Na in the streamwater is Ca-containing plagioclase rather than potassium feldspar. On the other hand, the average molar Ca/Na ratio of granite BDC in streamwater (0.47) is close to that of the bulk granite (0.32) and plagioclase (0.2 to 1.0; Anma et al. 1998). These data suggest that plagioclase is selectively weathered and releases Ca and Na into the water (Berner and Berner 1987). The average molar Mg/Na ratio in the granite BDC (0.10) is lower than the average molar Ca/Na and K/Na ratios (0.32 and 0.91). This result is consistent with the modal ratio of biotite in the granite, which is less than 0.05, and little Mg moves into secondary minerals (e.g., chlorite, vermiculite) during the chemical weathering of biotite (Nakano et al. 1991).

#### Fraction of Rain-Derived Sr in the Yakushima Streamwater

Similarly to the other cations, Sr in the streamwater is derived from both rainwater and the watershed’s bedrock. We used the following equation for the Sr isotope ratio to determine the proportions of the Sr derived from rainwater and BDC:

2 $$ {\left( {{{{^{{{87}}}{\text{Sr}}}} \left/ {{^{{{86}}}{\text{Sr}}}} \right.}} \right)_{\text{streamwater}}} = {f_{\text{Sr-rain}}}{\left( {{{{^{{{87}}}{\text{Sr}}}} \left/ {{^{{{86}}}{\text{Sr}}}} \right.}} \right)_{\text{rainwater}}} + \left( {{1} - {f_{\text{Sr-rain}}}} \right){\left( {{{{^{{{87}}}{\text{Sr}}}} \left/ {{^{{{86}}}{\text{Sr}}}} \right.}} \right)_{\text{BDC}}} $$

where *f*_Sr-rain_ is the ratio of the Sr in rainwater to that in streamwater.

The ^87^Sr/^86^Sr ratio in streamwater in the granite watersheds ranges from 0.70818 to 0.70876, whereas that in the Kumage sedimentary rock watersheds ranges from 0.71023 to 0.71345 (Fig. 4). This difference corresponds to the difference in the ratios for granite (0.70810 to 0.70827) and for the Kumage sedimentary rocks (0.71504 to 0.71606), indicating that part of the Sr in streamwater is derived from weathering of the bedrock in the watershed. Nevertheless, there is no altitudinal change in the ^87^Sr/^86^Sr ratio in Yakushima streamwater in the granite watershed (Fig. 4). This result suggests that the contribution of Sr from granite to the streamwater is close to that from the atmosphere. In contrast, Nakano et al. (2000) have shown that rain is the dominant source of seawater-derived Sr on Yakushima, with a uniform ^87^Sr/^86^Sr value of 0.70918 (Faure and Mensing 2005). However, it is difficult to determine the ^87^Sr/^86^Sr ratios for granite BDC and Kumage BDC, as these ratios are a function of factors such as the degree of chemical weathering, the Sr contents and ^87^Sr/^86^Sr ratios of the primary minerals, and the Sr content of the secondary minerals.

The initial ^87^Sr/^86^Sr ratio of the Yakushima granites at a formation age of 14 Ma was calculated to average 0.70760 (Table 3). The ^87^Sr/^86^Sr ratio of plagioclase and apatite in the granite was also estimated to be around 0.70760, since radiogenic Sr released by the decay of ^87^Rb is negligible in these minerals, which generally have a low Rb/Sr ratio, and because of the relatively young age of the Yakushima granite. If the ^87^Sr/^86^Sr ratio of granite BDC in the streamwater is identical to that of the bulk granite or plagioclase (plus apatite and carbonates), the value of *f*_Sr-rain_ can be calculated as 0.28 or 0.53, respectively. The former value is very close to the value of the rainwater proportion for Ca (*f*_Ca-rain_ = 0.32), which was determined by using Cl (as described in Section 3.1.3), but the plagioclase-based value is higher.

To determine the value of *f*_Sr-rain_ on the basis of the method using Cl, we estimated the Sr/Cl ratio of rainwater by using the following equation:

3 $$ {\left( {{{\text{Sr}} \left/ {\text{Cl}} \right.}} \right)_{\text{rain}}} = \left( {{{{{\text{S}}{{\text{r}}_{\text{sea-salt}}}}} \left/ {{{\text{C}}{{\text{l}}_{\text{rain}}}}} \right.}} \right) + \left( {{{{{\text{S}}{{\text{r}}_{\text{NSS}}}}} \left/ {{{\text{C}}{{\text{l}}_{\text{rain}}}}} \right.}} \right) $$

This equation can be expressed by using the proportion of NSS Sr in the rain (*f*_NSSSr-rain_), as follows:

4 $$ {\left( {{{\text{Sr}} \left/ {\text{Cl}} \right.}} \right)_{\text{rain}}} = {\left( {{{\text{Sr}} \left/ {\text{Cl}} \right.}} \right)_{\text{seawater}}} + \left[ {{{{{f_{\text{NSSSr-rain}}}}} \left/ {{\left( {{1 } - {f_{\text{NSSSr-rain}}}} \right)}} \right.}} \right]{\left( {{{\text{Sr}} \left/ {\text{Cl}} \right.}} \right)_{\text{seawater}}} $$

Although there is no report on the Sr content of Yakushima rain, the good correlation between the concentrations of Sr and Ca in the rain (*r* ^2^ = 0.88) at five sites in Japan indicates that it is possible to estimate the value of *f*_NSS Sr-rain_ by using the relationship between NSS Sr/Sr and NSS Ca/Ca, which was reported by Nakano et al. (2006). As the average NSS Ca/Ca (weight) of the Yakushima rain was reported to be 0.6 (Nakano et al. 2000), the *f*_NSS Sr-rain_ can be estimated to be 0.15 using Fig. 4 of Nakano et al. (2006). Accordingly, the molar ratio of Sr/Cl for Yakushima’s rain can be estimated to be 0.00019 by using Eq. 4 and the concentrations of Sr and Cl in seawater (Berner and Berner 1987). Because the Sr/Cl value in Yakushima’s streamwater (mean molar ratio for granite and sedimentary bedrock = 0.00057; Table 2) does not differ greatly between the granite and Kumage sedimentary rock watersheds, the rainwater proportion for Sr in the streamwater can be calculated to be 0.33 from Eq. 1. This value is close to the value of 0.28 obtained from Eq. 2 when the ^87^Sr/^86^Sr ratio of granite BDC is assumed to be the same as that of bulk granite rather than the result of selective leaching of cations from plagioclase and apatite. This result is consistent with that of Flanklyn et al. (1991), who reported that the ^87^Sr/^86^Sr ratio of shallow groundwater in Canada was identical to that of granite within the same watershed. However, the Sr isotopic coincidence between granite BDC and bulk granite is not well explained at present, and possible reasons for this coincidence must be examined in a future study.

The major source of Sr in the water of watersheds underlain by igneous rock is attributed to plagioclase with accessory apatite and carbonates because of its high Sr content and its high susceptibility to chemical weathering. Nevertheless, the presence of bedrock-derived K (64 %) and Mg (17 %) in the Yakushima streamwater indicates that potassium feldspar and biotite, despite their small Sr contributions, can still supply enough Sr into the streamwater to increase its ^87^Sr/^86^Sr ratio. Thus, the contributions of rainwater and watershed bedrock to the streamwater can be evaluated by comparing their elemental compositions and their ^87^Sr/^86^Sr ratios.

Figure 5 shows that the ^87^Sr/^86^Sr ratio of streamwater in the granite watershed does not differ significantly between the northwestern and southeastern sides, indicating that the chemical weathering of granite is not always intense in the northwestern side where the deposition of NSS SO_4_ is high. This result is consistent with the above-mentioned view that anthropogenic acid deposition of NSS SO_4_ from the Asian continent is not a major factor for the altitudinal decrease of pH in Yakushima streamwater. Residence time of rainwater would also become short with elevation, which leads to the generation of streamwater with low pH and dissolved ions. In order to evaluate the impact of acidic rain on Yakushima’s aquatic system, it will be necessary to monitor rainfall pH and the quality of streamwater at several sites with different elevations; currently, no such data are available for Yakushima Island.

### Soil–Vegetation System

#### Contribution of Atmospheric Sr to the Soil–Vegetation System

Similar to the situations with other elements, the Sr in terrestrial plants is ultimately derived from the atmosphere and the bedrock. In contrast with the ratios of many stable isotopes such as Si and Ca, the ^87^Sr/^86^Sr ratio in the TIMS analysis was determined after correcting for mass fractionation (Faure and Mensing 2005), so the plant Sr should have the same ^87^Sr/^86^Sr ratio as the ratio in the ambient soil solution. Given the different Sr isotope ratios in the two sources, it is possible to estimate their relative contribution to Sr in plants by comparing the variation in ^87^Sr/^86^Sr ratios and using the calculation method proposed by Graustein (1988). This method approximates the values as follows:

5 $$ {\left( {{{{^{{{87}}}{\text{Sr}}}} \left/ {{^{{{86}}}{\text{Sr}}}} \right.}} \right)_{\text{plant}}} = {f_{\text{Sr rain}}}{\left( {{{{^{{{87}}}{\text{Sr}}}} \left/ {{^{{{86}}}{\text{Sr}}}} \right.}} \right)_{\text{rainwater}}} + \left( {{1 } - {f_{\text{Sr rain}}}} \right){\left( {{{{^{{{87}}}{\text{Sr}}}} \left/ {{^{{{86}}}{\text{Sr}}}} \right.}} \right)_{\text{BDC}}} $$

Graustein (1988) estimated that about 60 to 80 % of the Sr in spruce and aspen in the Tesuque watershed of New Mexico is derived from precipitation. Likewise, Miller et al. (1993) reported that about 53 % of the Sr in the vegetation in a forest ecosystem in the Adirondack Mountains of New York was of atmospheric origin.

The ^87^Sr/^86^Sr ratios for terrestrial plants on Yakushima depend on the underlying bedrock. They ranged from 0.70861 to 0.70912 (mean = 0.70892) on granitic bedrock and from 0.70918 to 0.70935 (mean = 0.70925) on the Kumage sedimentary bedrock (Nakano et al. 2000). On Yakushima, the ^87^Sr/^86^Sr ratio in rainwater is close to that in seawater (Nakano et al. 2000). On the other hand, it is reasonable to assume that the chemical composition and Sr isotope ratio of BDC in soil water are similar to those in streamwater; in the granite watersheds, the ^87^Sr/^86^Sr ratio of BDC can be represented by the average ratio for Yakushima granite (0.70818). If these assumptions are valid, the proportion of rain-derived Sr in the plants on granite substrates would range from 43 to 94 % (mean = 74 %), indicating a large contribution of rain-derived Sr in Yakushima’s plants on a granite substrate.

The ^87^Sr/^86^Sr ratio of Kumage BDC is not easily evaluated compared with that of the granite BDC, because the Sr contents and ^87^Sr/^86^Sr ratios of the constituent minerals in the Kumage sedimentary rocks are more heterogeneous. However, if the *f*_Sr rain_ in streamwater of the Kumage sedimentary rock watersheds is the same as that in the granite watersheds (33 %), the ^87^Sr/^86^Sr ratio of Kumage BDC can be calculated as 0.71368 by using Eq. 2. By substituting this value into Eq. 5, the proportion of rain-derived Sr in the plants growing on the Kumage sedimentary substrate can be calculated to be more than 96 %. This value increases to 98 % if the Kumage BDC has the average ^87^Sr/^86^Sr ratio of the Kumage sedimentary rocks (0.71552). The dominance of rainwater sources of Sr in the plants growing in the Kumage sedimentary rock watersheds can likely be ascribed to the fact that vegetation in these watersheds is affected strongly by sea salt particles, as these sites are found mainly at lower elevations near the coast. Thus, plants on Yakushima are more enriched in rainwater-derived Sr of sea salt origin than streamwater, regardless of the bedrock type.

**Fig. 6:**
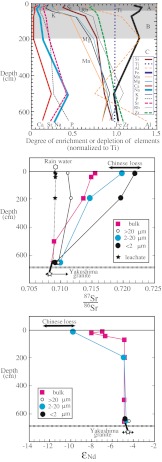
*Top*: profiles of the degree of enrichment and depletion of the soil elements (relative to a normalized value of 1.0 in the granite parent material, represented by the *dashed line*) as a function of depth in the soil at the Yotsuse site. *Middle*: profiles of the ^87^Sr/^86^Sr ratio and (*bottom*) of the *ε*_Nd_ values in the soil (for three particle size classes) as a function of depth and in the soil leachate. The C horizon is composed of weathered granite

Table 4 shows the soil chemical composition, ^87^Sr/^86^Sr ratio of plants, ^87^Sr/^86^Sr ratio, and *ε*_Nd_ value of soils at the Yotsuse site, which overlies a granite substrate. At this site, the three plant species that we sampled had similar ^87^Sr/^86^Sr ratios (0.70911 for the leaves of Japanese cedar, 0.70913 for plants, and 0.70915 for the leaves of Japanese red pine). These values were close to the ^87^Sr/^86^Sr ratio of seawater. From Eq. 5, we can calculate that 93 to 97 % of the Sr in the Yotsuse plants was derived from sea salt Sr. It is notable that the ^87^Sr/^86^Sr ratio in the plants is close to that of the exchangeable soil pool at different depths (which ranges from 0.70911 to 0.70920), with the exception of the lower C horizon, where the ^87^Sr/^86^Sr ratio (0.70884) is close to that of the underlying granite (Fig. 6). Another notable feature is that the ^87^Sr/^86^Sr ratio of the bulk soil at the Yotsuse site decreases from 0.71552 near the surface to 0.70908 with increasing depth. This ratio is generally higher and more variable than the ^87^Sr/^86^Sr ratios in the plants and in the exchangeable soil pool. The relationship between the Sr isotope ratios in the plants, soil, and exchangeable soil pool is consistent with the view that Sr and other nutrients in Yakushima’s plants are affected strongly by rainwater and are exchanged with the exchangeable soil pool rather than with the associated soil minerals.

**Table 4:** Elemental compositions and Sr and Nd isotopic ratios of soil and Sr isotopic ratios of plants at the Yotsuse site

Soil type	No.	Depth from surface	Si	Ti	Al	Fe	Mn	Mg	Ca	Na	K	P	Rb	Sr	Zr	
			wt%									ppm				
A horizon	A0	10 cm	26.5	0.63	10.3	13.8	0.03	0.58	0.21	0.45	1.56	209	109	57	189	
	A1	20 cm	25.8	0.65	11.2	15.0	0.03	0.62	0.11	0.35	1.47	127	103	53	187	
B horizon	A2	40 cm	26.5	0.54	11.3	15.1	0.03	0.52	0.11	0.36	1.74	105	110	47	170	
	A3	70 cm	27.7	0.41	11.3	15.2	0.03	0.50	0.05	0.36	2.82	105	143	40	134	
	A4	190 cm	28.1	0.34	11.6	15.5	0.03	0.46	0.04	0.39	3.14	140	158	42	122	
C horizon	A5	500 cm	33.5	0.36	7.1	9.5	0.05	0.67	0.42	1.36	4.43	179	208	89	150	
	A6	650 cm	30.6	0.30	10.6	14.3	0.04	0.47	0.19	0.70	3.30	249	186	40	135	
Soil type	No.	Depth from surface	Bulk			>20 μm			2–20 μm			<2 μm			1 N NH_4_Cl	H_2_O_2_
			^87^Sr/^86^Sr	^143^Nd/^144^Nd	*ε*_Nd_	^87^Sr/^86^Sr	^143^Nd/^144^Nd	*ε*_Nd_	^87^Sr/^86^Sr	^143^Nd/^144^Nd	*ε*_Nd_	^87^Sr/^86^Sr	^143^Nd/^144^Nd	*ε*_Nd_	^87^Sr/^86^Sr	^87^Sr/^86^Sr
A horizon	A0	10 cm	0.715521	0.512279	−7.00	0.711247			0.719919	0.512138	−9.76	0.721750			0.709105	0.709118
	A1	20 cm		0.512224	−8.08										0.709191	0.709055
B horizon	A2	40 cm	0.714919	0.512297	−6.65										0.709198	0.709196
	A3	70 cm		0.512388	−4.88										0.709168	
	A4	190 cm	0.713594	0.512386	−4.91	0.711721	0.512386	−4.92	0.714722	0.512386	−4.92	0.719593			0.709168	0.709223
C horizon	A5	500 cm	0.709079	0.512387	−4.90											
	A6	650 cm		0.512388	−4.88	0.709644	0.512398	−4.68	0.709990	0.512398	−4.68	0.709211	0.512420	−4.26	0.708839	0.709446
Plant																
Leaves of pine			0.709149													
Plants			0.709125													
Leaves of Japanese cedar			0.709105

#### Asian Dust Minerals in the Soil

The depth profile of elements in the soil column at Yotsuse differed among the elements (Table 4, Fig. 6). Ti and Zr are assumed to be immobile in the soil environment during the weathering process (Kirkwood and Nesbitt 1991). The degree of enrichment and depletion of element *x* in the soil at this site, termed *fx*, was calculated by using the following equation:

6 $$ fx = {{{\left( {{{{W_{\text{soil}}^x}} \left/ {{W_{\text{soil}}^{\text{Ti}}}} \right.}} \right)}} \left/ {{\left( {{{{W_{\text{gr}}^x}} \left/ {{W_{\text{gr}}^{\text{Ti}}}} \right.}} \right)}} \right.} $$

where $W_{\text{soil}}^x$ and $W_{\text{soil}}^{\text{Ti}}$ are the concentrations of *x* and Ti, respectively, in the soil and $W_{\text{gr}}^x$ and $W_{\text{gr}}^{\text{Ti}}$ are the corresponding concentrations in granite.

Most elements except Fe were depleted in the soil column, but the depletion pattern depended on the element (Fig. 6). Ca, Na, P, and Sr were depleted at all depths in the soil compared with their values in the granite parent material. The Ca content in the A and B horizons declined to less than 10 % of the value in the parent material, and the Na, Sr, and P contents in the A and B horizons declined to less than 20 % of the level in the parent material. On the other hand, the degree of depletion was less than 50 % for K, Mg, Mn, and Rb, and the magnitude of the depletion decreased with increasing depth in the C horizon owing to the weak degree of chemical weathering of minerals containing these elements at these depths. This pattern shows that plagioclase and small amounts of apatite and carbonates (White et al. 2005; Hartmann 2009) are more intensely weathered than potassium feldspar and biotite.

This result is consistent with the results from soil columns at two other sites on Yakushima (Shiratani-Unsuikyo and Shin-Takatsuka; Fig. 1). At all sites, the depletion of Ca, Na, P, and Sr in the soil compared with the levels in the original granite was large (generally >50 %), whereas the depletion was weak (10 to 50 %) for K, Si, Mg, Mn, and Rb. The depletion was largest for Ca, reaching more than 95 % in the B horizon at the Yotsuse and Shiratani-Unsuikyo sites. Soils in the C horizon are composed of primary and secondary minerals, and the original granite texture is well preserved. Accordingly, the depth variation of elements in the C horizon can be ascribed to chemical weathering, whereas the variations in the A and B horizons can likely be ascribed to the presence of organic matter and exotic materials in addition to the substrate materials.

The ^87^Sr/^86^Sr ratio and *ε*_Nd_ values of the C horizon at Yotsuse were independent of the particle size, and ranged from 0.70908 in the upper C horizon to 0.70999 and from −4.90 to −4.26, respectively. These *ε*_Nd_ values are indistinguishable from those of the Yakushima main granite (−5.3 to −4.1; Anma et al. 1998), whereas the ^87^Sr/^86^Sr ratios in the C horizon are higher than those of the Yakushima main granite (0.70818). This increased ^87^Sr/^86^Sr in the C horizon seems attributable to the enrichment by potassium feldspar, muscovite, and secondary minerals from biotite with high ^87^Sr/^86^Sr ratios. One notable feature is that the ^87^Sr/^86^Sr ratio in the bulk soil at Yotsuse tends to increase towards the surface, moving from the B horizon to the A horizon, whereas the *ε*_Nd_ value tends to decrease. This result indicates that the Yotsuse soil contains minerals that did not originate in the parent material, with a high ^87^Sr/^86^Sr ratio and a low *ε*_Nd_ value, and their contribution increases toward the surface of the soil column.

China’s Central Loess Plateau is a major depositional area for dust minerals emitted from the upwind desert areas of northern China and southern Mongolia. Loess in this region is composed of minerals produced by wind erosion and by evaporation (mainly calcite but also anhydrites and halides) that can be dissolved by water and acetic acid, phosphates and Mg-containing chlorites that can be dissolved by hydrochloric acid (HCl), and silicates and small amounts of oxides that resist chemical weathering. According to Yokoo et al. (2004), these minerals have distinct ^87^Sr/^86^Sr ratios, with values of 0.7111 ± 0.0004 (mean ± SD) for evaporite minerals, 0.7141 ± 0.0004 for phosphates and chlorites, and 0.7195 ± 0.0010 for silicates, whereas silicates have Nd isotope ratios similar to those of the bulk loess. According to Takahashi et al. (2001), the pH(H_2_O) value of forest surface soil at 1,034 sites in Japan ranged from 3.5 to 8.1, with a median of 5.1. Because the first two types of minerals are not stable in Japan’s acidic soils, it is possible that the acid-resistant silicate minerals from the Central Loess Plateau are still present in the soil of Yakushima Island.

Another notable feature is that the ^87^Sr/^86^Sr ratios and *ε*_Nd_ values of the Yotsuse soil depended on the particle size (Table 4, Fig. 6). In the A and B horizons, the ^87^Sr/^86^Sr ratio increases from 0.71125 to 0.71172 in the coarsest particles (>20 μm) to 0.71959–0.72175 in the finest particles (<2 μm). The particles >20 μm have a relatively constant ^87^Sr/^86^Sr ratio, irrespective of depth, indicating that they are derived from the granite substrate. The ^87^Sr/^86^Sr ratio (0.71992–0.72175) and *ε*_Nd_ value (−9.76) of the two fine-grained fractions (2–20 and <2 μm) in the upper A horizon are similar to those of acid-insoluble minerals in the loess of the Central Loess Plateau (average, 0.72063 and −12.5). Because the average diameter of Asian dust particles falling on Japan is about 4 μm (Nagoya University 1991), and because larger particles are less likely to be transported over long distances, the contribution of Asian dust would increase with decreasing particle size. The dependence of the Sr and Nd isotope ratios on particle size and depth in the Yotsuse soil column strongly suggests that the exotic minerals in the soil originated from silicates present in Asian dust and that the proportion of this foreign dust increases in the upper soil horizons.

**Fig. 7:**
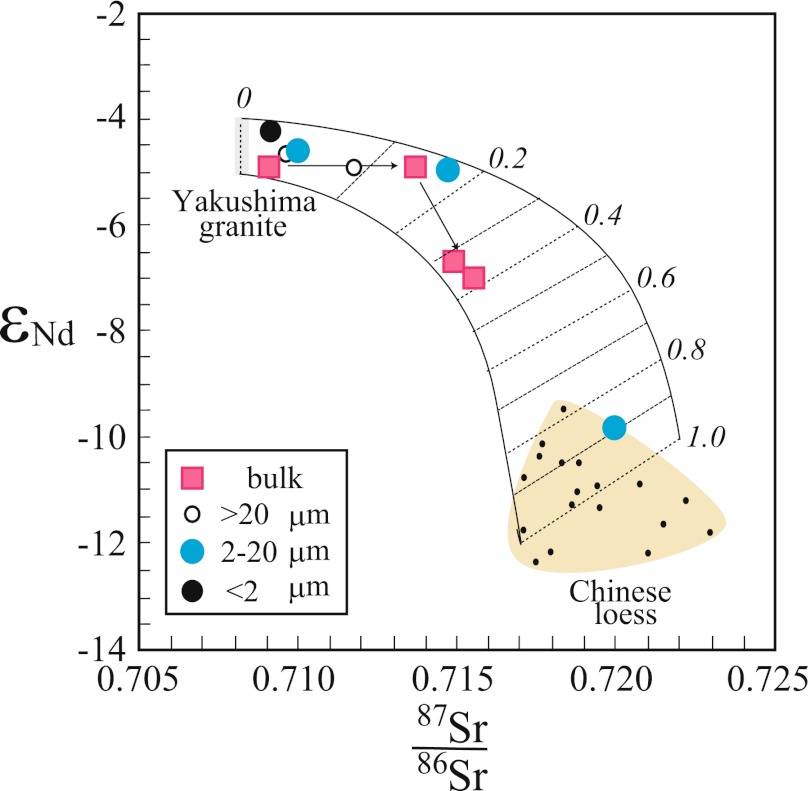
Plot of ^87^Sr/^86^Sr versus *ε*_Nd_ for soil minerals with three particle sizes and for bulk soil at the Yotsuse site. Data for the Chinese loess are from Nakano et al. (2004) for HCl residual minerals of surface soils from the Southern Gobi and Central Loess Plateau. Data for the Yakushima granite are those of Anma et al. (1998)

We evaluated the granite-derived and dust-derived minerals in the Yotsuse soil by using the concentrations and isotope ratios of Sr and Nd in the Yakushima granite and in Chinese loess silicates (Fig. 7). We assumed two different ^87^Sr/^86^Sr ratios and *ε*_Nd_ values for each of these components on the basis of the observed variation for the Chinese loess silicates and for the Yakushima granites and weathered silicates. The Sr and Nd contents of the dust minerals were assumed to be 150 and 20 ppm, respectively, whereas those of the granite-derived components were assumed to be 30 and 26 ppm, respectively, because the Sr content of the weathered granite is significantly lower than that of the granite parent material. On the basis of these assumptions, the ^87^Sr/^86^Sr ratios and *ε*_Nd_ values for the bulk soil and soil minerals with different particle sizes fell within a region between two lines that connect the range of values for the two components. Figure 7 shows that the contribution of Asian dust silicates ranges from 10 to 30 % in the B horizon and 40 % in the A horizon.

The concentration of Ca in silicates from the Chinese loess is significantly lower than that in the bulk loess, because Ca in the loess is mainly present in calcium carbonate (Table 3). Because carbonates dissolve easily in acidic rain but loess silicates, which have a low Ca content, are acid insoluble, it is likely that the Asian dust silicates do not play an important role in the biogeochemical Ca cycle of the Yakushima soil–vegetation system. In contrast, the concentrations of P in the bulk loess and carbonate-free (acetic acid insoluble) minerals were 818 and 591 ppm, respectively, versus only 108 ppm in the silicate (hydrochloric acid insoluble) minerals (Table 3). Because apatite will dissolve only in an acid solution stronger than that required to dissolve calcium carbonate (Jones et al. 1998), it does not dissolve substantially at the pH found in acidic rain. Most phosphorus in the Chinese loess (>80 %) is present as phosphates. Phosphorus in Asian dust plays a dominant role in regulating vegetation growth on base-poor soils that have undergone chemical weathering (Chadwick et al. 1999; Hartmann et al. 2008). It is noteworthy that there are no phosphate minerals in the upper Yotsuse soil. Accordingly, as long as phosphates in the Asian dust are more insoluble in acidic rain than carbonates during their transport through the atmosphere, the phosphates are likely to be dissolved in acidic soil and become the major source of P in the Yakushima soil–vegetation system.

#### Impacts of Atmosphere-Derived Materials on Yakushima Island

The unique topography of Yakushima, with jagged mountains facing the coast, provides favorable conditions for the formation of clouds from water vapor evaporated from the surrounding seawater and sea salt particles carried into the air by strong winds; this water vapor often condenses to form rain as a result of cooling as it rises along the mountain slopes. This process seems to be the primary control on the chemical composition of rainwater and streamwater, which resembles the composition of diluted seawater. It is considered that the water that originates in the sea is not acidic, but is instead somewhat alkaline owing to its high content of sea salts. However, rain is primarily acidic due to carbonic acid formed by dissolving CO_2_ in the atmosphere. It becomes more acidic by incorporation of anthropogenic SOx and NOx from the Asian continent, resulting in the formation of Yakushima acidic rain with pH values similar to those measured in other areas of Japan. Large amounts of acidic rain formed in this manner on Yakushima Island are favorable for the generation of acidic soil due to carbonic and organic acids formed in the soil–vegetation system. This geochemical process may accelerate chemical weathering of the Yakushima rocks, leading to leaching of Ca, Na, and Sr from plagioclase and of Ca and P from apatite, and depletion of these elements in the soil compared with levels in the parent materials.

However, the low concentrations of Ca, Sr, and HCO_3_ and the high concentrations of H^+^ in streamwater suggest that chemical weathering (mainly of plagioclase) is not sufficiently fast to compensate for the overload of H^+^ from atmospheric and pedogenetic inputs. Because the rainwater on Yakushima has a low K content, streamwater K is predominantly of bedrock origin. Despite its high resistance to chemical weathering, alkali feldspar is a major component of the bedrock and is thus a main source of K that is leached into the streamwater. Because Ca and P are depleted in the soil owing to selective weathering of Ca-containing minerals, both elements in the water are derived mainly from atmospheric deposition. The rain is low in Ca because of the dominant sea salt component. Accordingly, when Asian dust minerals interact with acidic rain before they arrive on Yakushima, calcium carbonate would dissolve into the acidic rain.

Apatite is a trace mineral and is more acid insoluble than carbonates. Because the apatite is enriched in Ca and P, it is a promising nutrient source for the island’s vegetation. Primary production of terrestrial ecosystem is limited by nitrogen availability (Vitousek and Howarth 1991; Schlesinger 1997). Satake et al. (1998) suggested that nitrogen aerosols and gases such as NOx and NH_4_ from the Asian continent are potentially limiting nutrients for mountainous forests in Yakushima. The concentration of NOx in the atmosphere of China has recently been increasing owing to the country’s rapid economic growth, which has been accompanied by rapidly growing combustion of fossil fuels (Richter et al. 2005). In addition, Asian dust activity has also been increasing since the 1990s over eastern China, Korea, and Japan (Chun et al. 2001; Kurosaki and Mikami 2003). Asian dust is known to play an important role in the rainwater chemistry in northern China (Xu et al. 2009). Accordingly, monitoring the inputs of anthropogenic materials and those of dust minerals from the Asian continent will be indispensable for evaluating their impacts on the soil–vegetation system and on the aquatic ecosystems of Yakushima Island. Understanding these impacts is essential if we are to preserve this important world natural heritage site.

## Conclusions

Yakushima’s rainwater contains dissolved cations that are highly enriched in the sea salt component, as well as substantial amounts of anthropogenic S and N compounds that lower rainwater acidity. However, even though Yakushima’s rain has an average acidity similar to that of Japan as a whole, the island receives three to four times the total deposition of atmospheric H^+^ owing to the large amount of precipitation (4,000 to 8,000 mm/year). The substantial impact of acidic rainwater and of seawater on Yakushima’s ecosystems can be seen in the low pH, Ca, and HCO_3_ levels and the high sea salt component in the island’s streamwater. It can also be seen in the Sr isotope ratios of the soil water and of land plants, which are close to the marine values. Sr and Nd isotope data further suggest that the depletion of Ca in the exchangeable soil pool compared with values in the granitic parent materials is attributable to selective weathering of plagioclase and apatite and the recent accumulation of water- and acid-insoluble Asian dust silicates, and that these secondary minerals are not a vital part of the exchange of base cations with plants. Future monitoring of the geochemistry of streamwater, particularly regarding Ca losses, will be important to support preservation of the natural heritage site of Yakushima Island.
